# SARS-CoV-2 Serosurvey in Addis Ababa, Ethiopia

**DOI:** 10.4269/ajtmh.20-0816

**Published:** 2020-09-22

**Authors:** John H. Kempen, Aida Abashawl, Hilkiah K. Suga, Mesfin Nigussie Difabachew, Christopher J. Kempen, Melaku Tesfaye Debele, Abel A. Menkir, Maranatha T. Assefa, Eyob H. Asfaw, Leul B. Habtegabriel, Yohannes Sitotaw Addisie, Eric J. Nilles, Joseph C. Longenecker

**Affiliations:** 1 Department of Ophthalmology, Massachusetts Eye and Ear, Schepens Eye Research Institute, Harvard Medical School, Boston, Massachusetts;; 2 Massachusetts Consortium on Pathogen Readiness (MassCPR), Boston, Massachusetts;; 3 MyungSung Christian Medical Center (MCM) Eye Unit, MCM General Hospital, Addis Ababa, Ethiopia;; 4 MyungSung Medical School, Addis Ababa, Ethiopia;; 5 Berhan Public Health and Eye Care Consultancy, Addis Ababa, Ethiopia;; 6 International Clinical Laboratories, Addis Ababa, Ethiopia;; 7 Ethiopian Biotechnology Institute, Addis Ababa, Ethiopia;; 8 Department of Emergency Medicine, Brigham and Women’s Hospital, Harvard Medical School, Boston, Massachusetts;; 9 Department of Epidemiology, Faculty of Public Health, Faculty of Medicine, Kuwait University, Kuwait City, Kuwait

## Abstract

In a serosurvey of asymptomatic people from the general population recruited from a clinical laboratory in May 2020 in Addis Ababa, Ethiopia, three of 99 persons tested positive for SARS-CoV-2 IgG (3.0%, 95% binomial exact confidence interval: 0.6–8.6%). Taking into account pretest probability and the sampling scheme, the range of plausible population prevalence values was approximately 1.0–8.4%. These results suggest that a larger number of people have been infected than the counts detected by surveillance to date; nevertheless, the results suggest the large majority of the general population in Addis Ababa currently is susceptible to COVID-19.

The global COVID-19 pandemic caused by SARS-CoV-2 is causing both mortality/morbidity and collateral social and economic damage related to public panic and aggressive public policy measures to contain the disease worldwide.^1^ The epidemic appears to have taken hold much more slowly in sub-Saharan Africa than in most of the world.^2^ Antibody testing to evaluate the population proportion previously infected with SARS-CoV-2 has the potential to guide public policy but has not been reported so far for sub-Saharan Africa.

Because a large proportion of cases of COVID-19 are asymptomatic and because the elderly are disproportionately affected with severe and symptomatic disease, we hypothesized that a young population might have experienced an epidemic without having recognized it before COVID-19 become widely known. Such a prior epidemic might explain slower growth of the epidemic in sub-Saharan Africa than elsewhere. We hypothesized that a severe influenza-like illness was epidemic in Addis Ababa, Ethiopia, around the time COVID-19 was recognized, and might represent a prior epidemic. Because responses taken to contain COVID-19 prevent population-based sampling, and because of the urgency of the situation, we undertook a serological study of approximately 100 persons presenting to a laboratory for other reasons, and an additional group convalescent from the outbreak in November 2019/February 2020, to assess the extent of exposure of the population in Addis Ababa and indirectly assess whether this epidemic may have been attributable to SARS-CoV-2.

The study was conducted at International Clinical Laboratories in Addis Ababa, Ethiopia, which offers the Abbott IgG test run on the ARCHITECT platform with approval of the Ethiopian Food and Drug Administration to the general public. This test, which has received the European Conformitè Europëenne mark and an Emergency Use Authorization from the U.S. Food and Drug Administration, has been found to have 100% sensitivity and 99.9% specificity using the Abbott-determined index value cutoff of 1.4 in an independent study by the University of Washington conducted in Idaho, the United States.^3^ It has not yet been independently validated in Africa.

Subjects enrolled as part of the two groups met the following eligibility criteria: age 14 years or older, resident in Addis Ababa for all of November 2019–February 2020, and no travel outside Ethiopia since November 1, 2019. The following exclusion criteria also were absent: sore throat, runny nose, cough or difficulty breathing, and/or hospitalized or quarantined in the last 28 days; measured temperature > 99.6 Fahrenheit, resting heart rate > 100/minute, and/or resting respiratory rate ≥ 25/minute; incarceration for a crime; unwilling to participate; or unable to consent. The study was approved in advance by the institutional review boards of MyungSung Medical College (Addis Ababa, Ethiopia) and Partners Healthcare (Boston, MA), and the Addis Ababa Regional Health Bureau; subjects provided written informed consent in Amharic or English.

After training the study team, 99 subjects were recruited from the participating laboratory’s waiting room during May 18–21 inclusive; their characteristics are summarized in Table 1. Among these, three tested positive for SARS-CoV-2 IgG (3.0%, 95% binomial exact CI: 0.6–8.6%). None of the positive cases were taking medications; one had a chronic runny nose with no other symptoms. Taking into account the sampling scheme and the pretest probability (1.3% based on 1,923 positive results among 152,334 tests as of June 9, 2020^4^; the range of plausible values according to the method of Larremore and others^5^ is given in Figure 1). Forty-five Ethiopians recruited from the investigators’ network who recalled being sick with a COVID-compatible illness between November 1, 2019 and February 29, 2020 were recruited and enrolled on May 22–27, of whom one tested positive for SARS-CoV-2 IgG (2.2%, 95% CI: 0.1–12%).

**Table 1:** Characteristics of subjects (self-reported) who tested negative or positive for SARS-CoV-2 IgG in Addis Ababa, Ethiopia (May 2020)

	SARS-CoV-2 IgG	
	Negative	Positive
Female (%)	55	67
Age (years), mean	37.8	33.3
Ethiopian (%)	97	100
Total count of people at home, mean	3.8	3.7
Count of children at home, mean	0.97	1.0
Healthcare worker (%)	19.4	33.3
Hypertension (%)	12.9	0.0
Diabetes mellitus (%)	12.1	0.0
Obesity (%)	4.4	0.0
Lung disease (%)	2.2	0.0
Pregnant (among females) (%)	9.8	0.0
Smoker (%)	3.3	0.0
Any symptoms* (%)	24.7	33.3
Contact with confirmed COVID-19 case (%)	1.1	0.0
Subject thinks (s)he had COVID-19 before† (%)	1.2	0.0
Exposure to traveler‡ (%)	8.6	0.0

* Any of the following symptoms each month between November 2019 and March 2020 inclusive: fever > 37.7°C; feverish (did not measure); chills; headache, coughing, or sneezing (more than normal); sore throat; difficulty breathing; runny/stuffy nose; “feeling more tired than usual”; decreased appetite; body or muscle aches; joint pains; decreased sense of smell or taste; nausea; vomiting > 2 times; abdominal pain; and diarrhea. The subject with positive test result reported a runny/stuffy nose for all 5 months and no other symptoms.

† Nine subjects were “not sure,” including one with positive test result.

‡ Family member, friend, or coworker traveled outside Ethiopia between November 2019 and March 2020 inclusive.

**Figure 1.**
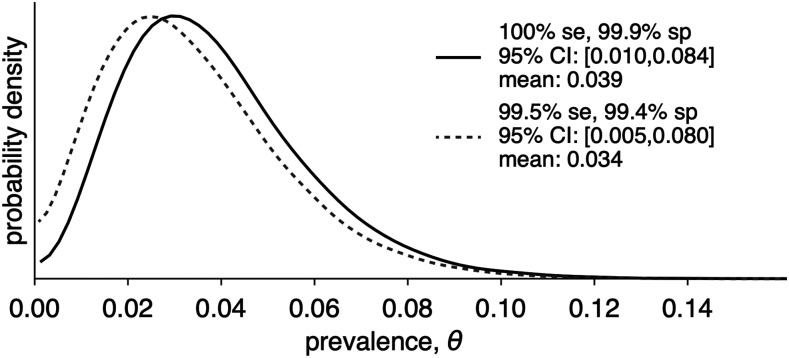
The range of plausible values of SARS CoV-2 IgG seropositivity among asymptomatic persons with no history of COVID-19 infection, expressed as probability density (Addis Ababa, Ethiopia, May 2020), based on sensitivity and specificity results for the test determined by the University of Washington^3^ is shown in the solid line. An alternative range under less favorable sensitivity and specificity assumptions is given in the dotted line. Se = sensitivity; Sp = specificity.

Our results—the first serological general population data on SARS-CoV-2 reported from sub-Saharan Africa—are a start toward the vast and important work of characterizing the extent of spread over time in this region, with approximately one-seventh of the world’s population. Although the results of this kind of sample are limited in scope and not easily generalizable, the results do suggest that the large majority of residents of Addis Ababa have not yet been infected by SARS-CoV-2 and are at risk. The results do not suggest any particular risk factors for SARS-CoV-2 seropositivity.

Decision-makers in Ethiopia, Africa, and elsewhere all are faced with the dilemma of weighing the trade-offs between the direct consequences of the COVID-19 epidemic and the health, economic, and other consequences of aggressive control measures.^2^ Given the very young age structure of sub-Saharan Africa with associated lesser direct risk from COVID-19,^6,7^ the risk of famine^8^ and limited access to essential services,^1^ as well as economic problems in this region as collateral problems from COVID-19–associated panic, and restrictive policies, different decisions may be appropriate in the African situation than in countries with an older population age structure and developed economic situation.^9^
